# Association between Activity Space Exposure to Food Establishments and Individual Risk of Overweight

**DOI:** 10.1371/journal.pone.0041418

**Published:** 2012-08-22

**Authors:** Yan Kestens, Alexandre Lebel, Basile Chaix, Christelle Clary, Mark Daniel, Robert Pampalon, Marius Theriault, S. V. p Subramanian

**Affiliations:** 1 Social and Preventive Medicine Department, Université de Montréal, Montreal, Quebec, Canada; 2 CRCHUM – Montreal University Hospital Research Centre, Montréal, Quebec, Canada; 3 Harvard School of Public Health, Boston, Massachusetts, United States of America; 4 INSERM – Pierre and Marie Curie University, Paris, France; 5 Samson Institute for Health Research, University of South Australia, Adelaide, Australia; 6 INSPQ, Quebec National Institute for Public Health, Quebec City, Quebec, Canada; 7 ESAD – Ecole Superieure d'Amenagement et Développement, Quebec City, Quebec, Canada; Universidad Peruana Cayetano Heredia, Peru

## Abstract

**Objective:**

Environmental exposure to food sources may underpin area level differences in individual risk for overweight. Place of residence is generally used to assess neighbourhood exposure. Yet, because people are mobile, multiple exposures should be accounted for to assess the relation between food environments and overweight. Unfortunately, mobility data is often missing from health surveys. We hereby test the feasibility of linking travel survey data with food listings to derive food store exposure predictors of overweight among health survey participants.

**Methods:**

Food environment exposure measures accounting for non-residential activity places (activity spaces) were computed and modelled in Montreal and Quebec City, Canada, using travel surveys and food store listings. Models were then used to predict activity space food exposures for 5,578 participants of the Canadian Community Health Survey. These food exposure estimates, accounting for daily mobility, were used to model self-reported overweight in a multilevel framework. Median Odd Ratios were used to assess the proportion of between-neighborhood variance explained by such food exposure predictors.

**Results:**

Estimates of food environment exposure accounting for both residential and non-residential destinations were significantly and more strongly associated with overweight than residential-only measures of exposure for men. For women, *residential* exposures were more strongly associated with overweight than non-residential exposures. In Montreal, adjusted models showed men in the highest quartile of exposure to food stores were at lesser risk of being overweight considering exposure to restaurants (OR = 0.36 [0.21–0.62]), fast food outlets (0.48 [0.30–0.79]), or corner stores (0.52 [0.35–0.78]). Conversely, men experiencing the highest proportion of restaurants being fast-food outlets were at higher risk of being overweight (2.07 [1.25–3.42]). Women experiencing higher residential exposures were at lower risk of overweight.

**Conclusion:**

Using residential neighbourhood food exposure measures may underestimate true exposure and observed associations. Using mobility data offers potential for deriving activity space exposure estimates in epidemiological models.

## Introduction

Towards better understanding the current obesity epidemic, there has been a concerted effort to examining the association between food environments that an individual is exposed to and their body mass index [1]. Studies have been looking at relations between food environments and food purchasing, diet, or more distal health outcomes like BMI, cardio-vascular outcomes or mortality [2], [3], [4]. But findings are mixed. For instance, association between fast food access and diet or BMI have been positive, negative or null [5], [6], [7].

The majority of studies on food environments and health have relied on measures of foodstore accessibility [8]. Some studies have considered more specific elements, such as food availability and costs [9], portion sizes [10], visual food cues, or availability of specific food types [11], [12], [13]. Geographic analyses of food environments generally use spatial proximity or density estimates to measure accessibility or exposure to foodstores [6]. Measures are established for point data such as postal codes or addresses, or for areal units, most often administratively defined and sometimes purposely designed, for example using ego-centered circular [14] or road-network buffers [15]. Proximity generally accounts for travel times or distance between the reference units and the closest foodstores [16]. Alternative accessibility measures based on gravity theory or space-time geography principles have more rarely been used [17]. Density measures are usually computed within a chosen areal unit by dividing the count of observation by the area or the population, or using kernel density estimation methods [18], [19].

In spatial epidemiology, the relationship between environmental exposures and individuals – and their corresponding health behaviors or disease outcome – is traditionally grounded to one reference location - most often, place of residence. Some have looked at exposure in non-residential locations such as schools [20], [21]. However, even then, the relation between access and health outcomes is assessed for one reference location only. A study integrating exposure to both residential and five non-residential regular activity places showed that ignoring non-residential exposures underestimated the association between residential exposure and self-reported health [22]. Another multi-location exposure study assessed the relation between BMI and accessibility to restaurants including fast food outlets around both home and the workplace [23]. No association was found for women, and for men, a significant inverse relation between BMI and restaurant proximity was found around workplaces only, and not around home.

Limiting measures of exposure to the local residential area may constitute a ‘local’ [24] or ‘residential’ [25] trap, and thus ignores actual ‘spatial polygamy’ [26], or the fact that we live and spatially relate to more than one ‘anchor point’, through a network of usual places [27]. Already in the 1950's, researchers in sociology and geography documented how daily activities included destinations outside of the residential neighbourhood [28]. The resulting multiple exposures may collectively influence health behaviors and health outcomes.

Current focus on residential areas is mainly due to the absence of data on people's activity destinations, at least in health surveys. Recent calls have been made to develop and test novel methodologies to collect such information, for example through web-based interactive mapping questionnaires that allow for precise collection of regular destinations or routes [29], or using wearable sensors such as Global Positioning Systems (GPS) devices. In a recent pilot study using such devices [30], collected tracks were used to derive activity space exposure to fast food outlets and supermarkets. Path area measures of exposure to fast food outlet were positively associated with dietary fat intake and negatively with whole grain intake. Whereas resorting to precise GPS data allows to show the potential importance of accounting for multiple exposures, use of GPS devices also presents some limitations, and has not yet been applied to large samples.

Alternatively, data on daily mobility are often collected in travel surveys, mainly developed for transportation planning purposes, or in other sources interested in specific aspects of mobility such as commuting. Such mobility information was recently used to estimate non-residential exposure to air pollutants in Vancouver and California [31]. Not accounting for non-residential exposure to NO^2^ underestimated the relative risk from 20 to 30% in Vancouver and 7% in California. Quite logically, this bias furthermore increased with distance and time spent away from home.

Mobility data are most often used to model travel behavior and to support land use and road network planning. But such data also showed that people with similar characteristics had similar exposure patterns to foodstores when their mobility was accounted for [32]. In continuity with these findings, we hypothesize that it is possible to use such mobility data to predict the *types* of places [33] people experience and, consequently, to better assess exposures to environmental determinants of health. This feasability study tests a novel method combining various datasets to assess activity space patterns of exposure to foodstores, and their relation to overweight. Travel surveys, foodstore listings and health surveys are combined using a GIS and modelling techniques. Models of multiple exposures to foodstores are developed and related associations with individual risk and local differences in overweight are tested in a multilevel framework [34]. The results of this feasability study have important implications relevant to multilevel policy and public health interventions which must target multiple settings to more effectively respond to the epidemic of overweight/obesity.

## Methods

### Data

#### Mobility data

Data from computer-assisted telephone interview travel surveys, conducted by the Quebec Ministry of Transport in 2003 in Montreal and in 2001 in Quebec City provided geographic coordinates of all activity location for one autumn weekday for all individuals surveyed. The survey methodology has been reported elsewhere [35]. Briefly, survey respondents were selected via random digit dialing. Interviews conducted by government employees lasted in average 11 minutes, and covered questions on household, household members, and all trips and activity destinations of all household members aged 4 and up. Reported activity locations were georeferenced through a GIS-powered survey application. All reported destinations for a given individual were used to derive measures of multiple exposures to places. Analysed data was restricted to adult participants living on the Montreal Island (n = 52,381) and in the Quebec City agglomeration (n = 45,718). We further selected mobile participants who had traveled to at least one non-residential destination (n = 41,252 and n = 36,768 respectively).

#### Foodstores typology

We geocoded all businesses and services from a private business registry obtained in 2005 (Tamec Inc.) located in the Montreal (n = 112,723) and Quebec Metropolitan Areas (n = 34,973). An on-site ground truthing study showed good validity of the foodstore registry [36]. Based on Standard Industrial Codes (SIC), we extracted corner stores, restaurants, fruit and vegetable stores, and supermarkets. Supermarkets and fruit and vegetable stores were combined into a unique category to represent foodstores offering access to fresh fruits and vegetables [37]. From the listing of restaurants, we further identified fast food outlets as places serving predominantly high-caloric food and relying primarily on self-service. Classification of fast food outlets was based on the restaurant name. Replication of the coding exercise three months later revealed high intra-rater reliability, with a kappa of 0.902 for Montreal and 0.960 for Quebec City.

#### Foodstore density

We transformed the point distribution of stores into continuous surfaces using kernel density estimations, with a quartic kernel and an adaptive bandwidth [19]. Kernel density determination is a recommended geographic method to establish accessibility or exposure measures to amenities in health research [38]. We further computed the ratio of densities of fast food outlets on all restaurants.

#### Neighborhood units

We used local health services units in Montreal (n = 29), and the current 36 neighborhoods of Quebec City, as well as two adjacent municipalities part of the urban continuum for which travel data was available (total n = 38).

#### Neighborhood characteristics

We extracted 2001 Census tract neighborhood socio-demographic and urban form data, and compiled average values within a neighborhood, weighted according to population. Urban form measures previously associated with mobility, that is, the density of four-way intersections, and an entropy index of land use mix integrating open areas, residential, industrial, and commercial land were computed [39]. Travel survey data was further used to compute a neighborhood-level measure of private vehicle accessibility, calculated as the average ratio of number of cars divided by the number of driving licenses in a given household.

#### Overweight

We used self-reported height and weight from participants of repeated cross-sectional cycles 2.1 (2003) and 3.1 (2005) of the Canadian Community Health Survey (CCHS) residing on the Montreal island and in the Quebec Area, based on geocoding of their 6-digit postal code. Some participants were interviewed by phone, others were met in person. Body mass index (BMI, in kg/m^2^) greater or equal to 25 was considered to define overweight. We excluded pregnant women, individuals being underweight (BMI<18.5), and those with an exceptionally high BMI (>70 kg/m^2^), or values for which socio-demographic neighborhood information was missing.

#### Individual, household, and Census tract data

Individual- and household-level variables available in both the travel surveys and the CCHS included age, gender, occupation, household type and household size. Census tract characteristics were obtained from Statistics Canada. Complementary data on income and educational attainment were further obtained for CCHS participants.

#### Ethics

This research was approved by the Montreal University Hospital Research Center Ethical review board. Written informed consent was obtained by Statistics Canada previous to survey administration among participants of CCHS. Travel survey participants, being interviewed through a Computer Assisted Telephone Interview (CATI), provided informed consent over the phone. The ethics committee approved both consent procedures.

### Modeling

**Figure 1:**
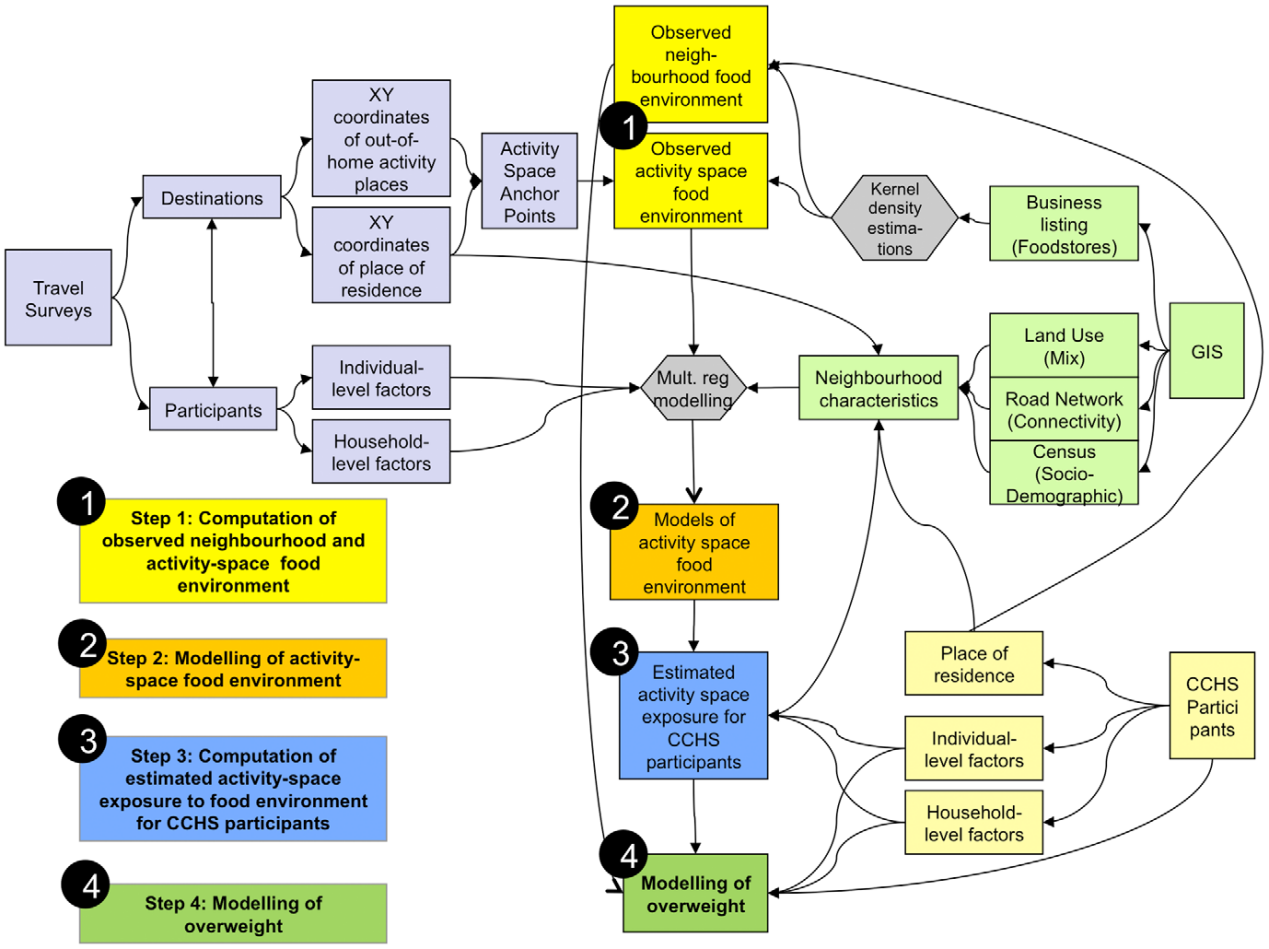
Synthetic view of database linkages and modelling steps.

The modeling procedure contains four steps (See Figure 1).

Step 1: Computation of Observed Activity Space Foodstore exposures: for each travel survey participant and for each foodstore type, the foodstore kernel densities measured at all participant's activity destinations were averaged.

Step 2: Modelling of observed activity space foodstore exposures: These measures where modelled in a multiple regression framework using individual, household, and place of residence characteristics. Forward stepwise regressions were used to establish models with the highest predictive power, for each foodstore type.

Step 3: Computation of two types of food environment exposures for health survey participants:. A ‘residential’ measure of foodstore exposure was computed using kernel densities of foodstores at place of residential. An ‘activity space’ measure was computed by applying the predictive model previously fitted with travel survey participants (step 3), resulting in *estimated* activity space foodstore exposures for CCHS participants

Step 4: Modelling of overweight: overweight was modelled in a multilevel logistic framework. For each store type, we could assess the association with overweight using either the observed residential or the estimated activity space foodstore exposure variable. It is important to note that observed residential measures are neighbourhood-level exposures, whereas estimated activity-space measures are individual-level exposures. These exposure predictors were divided in quartiles, the lowest quartile being used as reference category. For residential exposure, the 29 Montreal neighborhoods were divided in groups of 7, 7, 7 and 8 units, and in Quebec in groups of 9, 10, 10 and 9 units. The same inter-quartile ranges were used to establish quartiles of individual-level activity-space measures, so as to be able to compare coefficients for both type of predictors. For example, the highest quartile in Montreal contains 8/29*100 = 27.6% of observations [40].

The multilevel modeling followed a four-step scheme. Type 1 models provide variances estimates without covariate (null models). Type 2 models controlled for individual-level socio-demographic measures and neighborhood educational attainment. Type 3 models included observed neighborhood-level residential foodstore density predictors, which could then be compared to models integrating individual-level activity space foodstore density estimates (Type 4 models). All models were done with the residual iterated generalized least squares (RIGLS) and predictive quasi-likelihood (PQL) methods using MLwiN (Release 2.17). Sampling weights were deduced by normalizing CCHS population weights by taking the sample plan effect into account [41]. Models were stratified by city and gender. The Median Odd Ratios was estimated as the basis for interpreting between-neighborhood variance [42]. It can be conceptualised as the increased risk of being overweight (in median) when moving to an area with a higher risk. A MOR of one indicates no (residual) between-neighbourhood differences in the risk of overweight. Reductions in MOR compared to the null models provided information on the proportion of between-neighborhood variance being explained with Type 2, 3 and 4 models.

## Results

Table 1 shows that CCHS and travel survey samples were very comparable, both in Montreal and Quebec City. The analysis samples included 41,252 travel survey participants in Montreal (36,768 in Quebec City) and 3,244 CCHS participants (2,324 in Quebec City).

**Table 1:** Summary statistics for participants of the Travel Survey and Canadian Community Health Survey (CCHS), Montreal and Quebec City.

		Montreal				Quebec			
		Travel Survey		CCHS		Travel Survey		CCHS	
		n = 41,252		n = 3,244		n = 36,768		n = 2,334	
Variable	Variable Description	Mean	Stdd	Mean	Stdd	Mean	Stdd	Mean	Stdd
**OD/CCHS common variables**									
***Individual and household variables***									
Male	Being a male*	49,1%	50,0%	47,4%	49,3%	48,7%	50,0%	43,6%	53,2%
Age	Age of participant*	42,80	15,98	45,23	17,37	44,49	15,32	45,53	18,29
Age 18–24	Between 18 and 24 years old*	14,0%	34,7%	12,1%	32,6%	12,3%	32,8%	13,8%	37,0%
Age 25–44	Between 25 and 44 years old*	42,7%	49,5%	40,1%	48,9%	38,1%	48,6%	35,3%	51,3%
Age 45–64	Between 45 and 64 years old*	32,2%	46,7%	31,2%	46,2%	38,5%	48,7%	35,9%	51,5%
Age 65 up	More than 64 years old*	11,2%	31,5%	16,5%	37,0%	11,1%	31,4%	15,0%	38,4%
Household size	Number of persons in the household*	2,68	1,31	2,54	1,36	2,78	1,20	2,59	1,32
Couple with children	Household type: Couple with children*	25,7%	43,7%	35,8%	47,8%	34,6%	47,6%	37,3%	51,9%
Couple without children	Household type: Couple without children*	34,9%	47,7%	22,3%	41,5%	31,5%	46,4%	30,7%	49,5%
Single parent	Household type: Single parent*	3,0%	17,2%	7,5%	26,3%	3,0%	17,1%	5,9%	25,2%
Single person	Household type: Single person*	17,3%	37,8%	22,7%	41,8%	11,9%	32,4%	17,2%	40,5%
Other household	Other household type*	19,2%	39,3%	11,7%	32,1%	19,0%	39,3%	8,9%	30,6%
***Census Tract environmental predictors used to calibrate model of observed activity space exposure with OD participants and to estimate activity space exposure for CCHS participants***									
Land Use Mix	Entropy index of land use mix*	55,4%	16,1%	76,9%	7,7%	46,8%	19,6%	52,8%	22,1%
Connectivity	Number of four-ways intersections per square km*	29,82	21,54	25,28	13,45	17,63	27,64	14,08	20,17
1946 dwellings	Proportion of dwellings built before 1946	19,9%	21,5%	19,5%	16,1%	12,0%	16,7%	11,4%	16,3%
% single persons	Proportion of households of single persons*	42,6%	12,0%	43,2%	10,0%	44,5%	8,1%	44,3%	7,1%
% immigrants	Proportion of immigrants*	25,7%	13,8%	26,2%	11,9%	3,4%	2,4%	3,4%	2,2%
% 1-year movers	Proportion having moved last year*	16,1%	6,4%	16,4%	3,9%	14,4%	6,8%	14,3%	6,0%
% motorised households	Proportion of households with a car*	65,2%	16,9%	73,3%	10,6%	75,1%	11,5%	75,2%	13,1%
% less than 9th year	Proportion of people with educational attainment below 9th grade*	14,7%	8,4%	14,7%	6,9%	11,7%	6,8%	11,5%	6,4%
***Activity space exposure to food environment (Observed for travel survey participants, estimated for CCHS participants)***									
AS-COR	Activity space exposure to corner stores	4,14	3,07	4,16	2,06	1,26	0,92	1,15	0,70
AS-FV	Activity space exposure to stores offering fresh fruits and vegetables	1,18	0,89	1,12	0,50	0,47	0,31	0,42	0,20
AS-FFO	Activity space exposure to fast food outlets	4,04	6,74	2,02	1,30	1,41	1,05	1,24	0,60
AS-FSR	Activity space exposure to full-service restaurants	22,62	32,06	11,40	11,30	5,64	7,95	3,25	5,00
AS-FFP	Activity space exposure to proportion of restaurants being fast food outlets	0,19	0,07	0,19	0,04	0,32	0,14	0,33	0,08
**Additional CCHS-specific variables used to model overweight**									
***Individual and household***									
Overweight or obese****	BMI>25	-	-	45,4%	49,7%	-	-	43,8%	53,3%
Obese****	BMI>30	-	-	14,2%	34,9%	-	-	12,1%	35,1%
Less than 9th year	Educational attainment below 9th grade	-	-	20,0%	39,9%	-	-	14,5%	37,8%
College	Educational attainment college	-	-	50,9%	49,9%	-	-	62,1%	52,1%
University Degree	With university degree	-	-	29,1%	45,3%	-	-	23,5%	45,5%
Missing income	Income data incomplete	-	-	12,3%	32,8%	-	-	13,2%	36,3%
Lower income**	Lower income category	-	-	27,8%	44,8%	-	-	20,9%	32,3%
Higher Income	Higher income category	-	-	59,7%	49,0%	-	-	65,9%	50,4%
***Level-2 objective neighbourhood exposure to food environment measure*** ***									
O-NF COR	Observed nbhd exposure to corner stores	3,69	3,49	3,53	3,11	1,13	1,17	0,95	1,04
O-NF FV	Observed nbhd exposure to stores offering fresh fruits and vegetables	1,13	1,09	1,12	1,10	0,40	0,39	0,11	0,10
O-NF FFO	Observed nbhd exposure to fast food outlets	1,68	2,49	1,64	1,80	1,06	1,20	0,91	1,08
O-NF FSR	Observed nbhd exposure to full-service restaurants	10,75	20,06	9,84	13,82	3,47	7,86	2,85	6,99
O-NF FFP	Observed nbhd measure of the proportion of restaurants being fast food outlets	0,27	0,12	0,20	0,06	0,01	0,00	0,39	0,13

x Significant predictor of O-ASF.

* Predictors of activity space exposure.

** Household income < = $30 k for 1–2 person household, < = $40 k for 3–4 person; < = 60 k for 5 persons or more.

*** Data for OD participants shown for purpose of comparison only, not used in modelling procedure.

**** Provided for indicative purposes, not used in models.

OD: origin-destination travel survey; CCHS: Canadian community health survey; ASF: activity space foodscape exposure; O-NF: objective neighbourhood.

foodscape exposure; COR: corner stores; FVS: fruit and vegetable stores; FFO: fast food outlets; FSR: full service restaurants; FFP: proportion of restaurants being fast food outlets.

### Observed neighborhood and activity space foodstore exposures

Residential exposures to foodstores indicated overall higher experienced foodstore densities in Montreal than in Quebec City. For travel survey participants, the highest residential densities concerned full-service restaurants, followed by corner stores, fast food outlets and fruit and vegetable stores. Densities were systematically higher in Montreal. Residential exposures were similar for travel survey and CCHS participants in Montreal. In Quebec City, residential exposures were slightly lower among CCHS participants. Among travel survey participants and compared to residential exposures, observed activity space exposures were similar for corner stores and fruit and vegetable stores, but significantly higher for fast food outlets and full service restaurants, and the contrast between residential and activity space densities was stronger in Montreal than in Quebec City. More details on activity space foodstore exposures and comparison with residential exposures can be found elsewhere [32].

### Modelling of observed activity space exposures of travel survey participants

Adjusted R-squares for the modelling of observed activity space exposures varied in Montreal from 0.21 to 0.48 for exposure to fast food outlets and full service restaurants respectively, and in Quebec from 0.27 to 0.52 for the proportion of restaurants being fast food outlets and corner stores. Individual-, household and place of residence variables that were statistically significant predictors of activity space measures are flagged in Table 1.

### Computation of estimated activity space exposures for CCHS participants

Table 1 shows mean values and standard deviations of both travel survey observed and CCHS estimated activity space foodstore exposures were comparable.

### Multilevel modeling of overweight (See Table 2)

**Table 2:** Multilevel modeling of overweight, Canadian Community Health Survey, Cycles 2.1 & 3.1, Montreal and Quebec City, by gender.

		Montreal										Quebec									
		Men					Women					Men					Women				
1- Null model	**MOR**		**1,56**			*		**1,65**			*		**1,06**			*		**1,51**			*
2- Individual and neighbourhood SES model		**OR**	**(95% CI)**				**OR**	**(95% CI)**				**OR**	**(95% CI)**				**OR**	**(95% CI)**			
	Age 25–44	1,83	1,05	-	3,20	*	2,76	1,44	-	5,30	*	5,31	2,90	-	9,71	*	1,38	0,75	-	2,53	
	Age 45–64	2,60	1,37	-	4,94	*	5,02	2,75	-	9,14	*	6,91	3,87	-	12,34	*	2,15	1,24	-	3,73	*
	Age 65 up	2,23	1,26	-	3,94	*	4,66	2,26	-	9,59	*	4,64	2,27	-	9,46	*	1,93	0,95	-	3,93	
	Low income	0,75	0,49	-	1,15		1,08	0,77	-	1,50		0,81	0,55	-	1,19		1,19	0,82	-	1,73	
	Missing income	1,25	0,83	-	1,89		0,98	0,64	-	1,49		0,90	0,53	-	1,51		0,90	0,55	-	1,48	
	College or high school	0,98	0,74	-	1,29		1,43	0,99	-	2,06		1,47	1,07	-	2,02	*	0,94	0,50	-	1,76	
	Less than 9th grade	1,74	1,14	-	2,67	*	2,73	1,49	-	5,03	*	1,25	0,78	-	2,01		1,86	0,95	-	3,66	
	Nbhd % low education	1,01	0,81	-	1,25		1,15	0,93	-	1,42		0,98	0,88	-	1,08		1,24	1,06	-	1,45	*
	**MOR**		**1,55**		**−1,8%**			**1,50**		**−23,5%**			**1,00**		**−100%**			**1,46**		**−10,6%**	
3- Observed Neighbourhood Exposure models †		**OR**	**(95% CI)**				**OR**	**(95% CI)**				**OR**	**(95% CI)**				**OR**	**(95% CI)**			
	Corner stores Q2	0,65	0,40	-	1,06		0,71	0,45	-	1,11		0,79	0,57	-	1,07		0,71	0,47	-	1,07	
	Corner stores Q3	0,60	0,35	-	1,02		0,72	0,45	-	1,15		0,92	0,68	-	1,25		0,81	0,54	-	1,22	
	Corner stores Q4	0,49	0,30	-	0,79	*	0,51	0,37	-	0,71	*	0,70	0,54	-	0,91	*	0,54	0,32	-	0,91	*
	**MOR**		**1,48**		**−14,6%**			**1,39**		**−40,1%**	*		**1,00**		**−100%**			**1,44**		**−13,8%**	
	Fruit and vegetable stores Q2	0,70	0,43	-	1,14		0,74	0,47	-	1,15		0,74	0,52	-	1,05		0,86	0,58	-	1,27	
	Fruit and vegetable stores Q3	0,44	0,28	-	0,69	*	0,71	0,45	-	1,12		0,90	0,68	-	1,20		0,60	0,40	-	0,91	
	Fruit and vegetable stores Q4	0,65	0,38	-	1,09		0,49	0,34	-	0,71	*	0,76	0,62	-	0,92	*	0,59	0,37	-	0,93	*
	**MOR**		**1,45**		**−18,9%**			**1,40**		**−38,0%**	*		**1,00**		**−100%**			**1,40**		**−20,6%**	*
	Fast food outlets Q2	0,70	0,44	-	1,12		0,73	0,48	-	1,11		0,75	0,54	-	1,03		0,89	0,61	-	1,31	
	Fast food outlets Q3	0,61	0,37	-	1,02		0,63	0,46	-	0,88	*	0,92	0,67	-	1,27		0,65	0,41	-	1,03	
	Fast food outlets Q4	0,50	0,29	-	0,85	*	0,53	0,32	-	0,89	*	0,77	0,63	-	0,94	*	0,56	0,36	-	0,87	*
	**MOR**		**1,48**		**−14,6%**			**1,40**		**−38,9%**	*		**1,00**		**−100%**			**1,41**		**−19,9%**	*
	Full service restaurants Q2	0,62	0,40	-	0,95	*	0,75	0,48	-	1,17		0,70	0,54	-	0,91	*	1,07	0,72	-	1,60	
	Full service restaurants Q3	0,55	0,36	-	0,85	*	0,64	0,48	-	0,87	*	1,00	0,79	-	1,28		0,68	0,44	-	1,06	
	Full service restaurants Q4	0,46	0,29	-	0,74	*	0,54	0,32	-	0,90	*	0,66	0,55	-	0,80	*	0,63	0,40	-	1,00	*
	**MOR**		**1,43**		**−22,4%**			**1,40**		**−38,0%**			**1,00**		**−100%**			**1,41**		**−20,2%**	
	FFO/Restaurant proportion Q2	1,61	1,15	-	2,26	*	2,28	1,49	-	3,49	*	1,20	0,86	-	1,66		1,30	0,82	-	2,07	
	FFO/Restaurant proportion Q3	1,97	1,33	-	2,90	*	1,88	1,11	-	3,17	*	0,99	0,67	-	1,48		2,24	1,57	-	3,20	*
	FFO/Restaurant proportion Q4	2,09	1,41	-	3,10	*	1,80	1,10	-	2,95	*	1,14	0,88	-	1,48		1,51	1,02	-	2,24	*
	**MOR**		**1,45**		**−20,5%**			**1,38**		**−41,8%**	*		**1,00**		**−100%**			**1,36**		**−29,8%**	
4- Estimated Activity Space Food Exposure Models		**OR**	**(95% CI)**				**OR**	**(95% CI)**				**OR**	**(95% CI)**				**OR**	**(95% CI)**			
	Corner stores Q2	0,94	0,61	-	1,47		1,12	0,74	-	1,70		0,69	0,42	-	1,14		1,00	0,65	-	1,53	
	Corner stores Q3	0,90	0,58	-	1,39		1,01	0,66	-	1,54		0,61	0,43	-	0,87	*	0,82	0,50	-	1,32	
	Corner stores Q4	0,52	0,35	-	0,78	*	0,72	0,42	-	1,25		0,55	0,40	-	0,77	*	0,84	0,43	-	1,63	
	**MOR**		**1,44**		**−21,8%**			**1,45**		**−30,2%**			**1,00**		**−100%**			**1,45**		**−11,3%**	
	Fruit and vegetable stores Q2	1,07	0,73	-	1,55		0,91	0,64	-	1,31		0,60	0,45	-	0,81	*	0,89	0,57	-	1,39	
	Fruit and vegetable stores Q3	0,77	0,52	-	1,14		1,13	0,74	-	1,73		0,60	0,42	-	0,86	*	0,72	0,45	-	1,15	
	Fruit and vegetable stores Q4	0,70	0,45	-	1,09		0,68	0,40	-	1,16		0,68	0,49	-	0,94	*	0,77	0,44	-	1,33	
	**MOR**		**1,46**		**−17,4%**			**1,40**		**−37,7%**			**1,00**		**−100%**			**1,41**		**−19,5%**	
	Fast food outlets Q2	1,03	0,63	-	1,67		1,43	1,05	-	1,94	*	0,84	0,56	-	1,27		1,04	0,58	-	1,87	
	Fast food outlets Q3	0,66	0,40	-	1,10		1,00	0,61	-	1,65		0,71	0,48	-	1,04		1,02	0,57	-	1,81	
	Fast food outlets Q4	0,48	0,30	-	0,79	*	0,99	0,64	-	1,55		0,61	0,46	-	0,81	*	0,83	0,43	-	1,59	
	**MOR**		**1,39**		**−30,8%**			**1,50**		**−23,0%**			**1,00**		**−100%**			**1,49**		**−3,8%**	
	Full service restaurants Q2	0,76	0,44	-	1,33		1,09	0,75	-	1,59		0,76	0,52	-	1,11		1,31	0,95	-	1,81	
	Full service restaurants Q3	0,77	0,48	-	1,21		1,21	0,79	-	1,84		0,67	0,48	-	0,94		0,92	0,57	-	1,50	
	Full service restaurants Q4	0,36	0,21	-	0,62	*	0,88	0,53	-	1,46		0,69	0,49	-	0,97	*	0,85	0,51	-	1,41	
	**MOR**		**1,41**		**−26,0%**			**1,49**		**−24,8%**			**1,00**		**−100%**			**1,44**		**−14,5%**	
	FFO/Restaurant proportion Q2	1,45	0,86	-	2,45		1,69	1,00	-	2,84	*	1,43	0,99	-	2,06		1,09	0,62	-	1,94	
	FFO/Restaurant proportion Q3	1,66	0,94	-	2,92		1,51	0,81	-	2,83		1,32	0,98	-	1,77		1,15	0,69	-	1,92	
	FFO/Restaurant proportion Q4	2,07	1,25	-	3,42	*	2,05	1,30	-	3,22	*	1,45	0,92	-	2,29		1,27	0,75	-	2,17	
	**MOR**		**1,45**		**−19,6%**			**1,42**		**−35,1%**			**1,00**		**−100%**			**1,44**		**−13,1%**	

NB: Reference category: 1st Quartile of Exposure; MOR: Median Odd Ratio; OR: Odd Ratio; CI: Confidence Interval.

* Significant at p<0.05.

Type 1 (null) models: Between-neighborhood variance in the prevalence of overweight was statistically significant except among men in Quebec City. It was higher in Montreal – MOR of 1.56 for men and 1.65 for women –, than in Quebec City, were it was weaker for men (1.06) than for women (1.51). In both cities, the between-neighborhood variance was higher among women than among men.

Type 2 models: Introduction of individual level variables of age, income and both individual and neighborhood-level educational attainment reduced the MOR to 1.55 (−1.8%) and 1.50 (−23.5%) respectively for men and women in Montreal, and to 1.46 (−10.6%) for women in Quebec City. Between-neighborhood variance became null among men in Quebec City (MOR = 1).

Type 3 models: Level 2 variables of neighborhood exposure to foodstores were statistically significant in almost all cases. Living in the quartile of neighborhoods with the highest foodstore densities in comparison to the lowest was associated with a lower risk of being overweight (See Table 2). On contrary, living in a neighborhood with a higher proportion of restaurants being fast food outlets increases the risk of being overweight, except for males in Quebec City.

Type 4 models: In both cities, individual-level activity space foodstoreexposures to crude densities were significantly associated with overweight among men but not among women. Among males, activity space predictors were more strongly associated with overweight than residential predictors, leading to stronger decreases in MOR, whereas the contrary was true for females. Reductions in MOR were stronger when using activity space predictors than residential predictors, except for fruit and vegetable stores and the proportion of full service restaurants being fast food outlets, where both types of predictors conducted to similar reductions in unexplained area-level variance.

**Figure 2:**
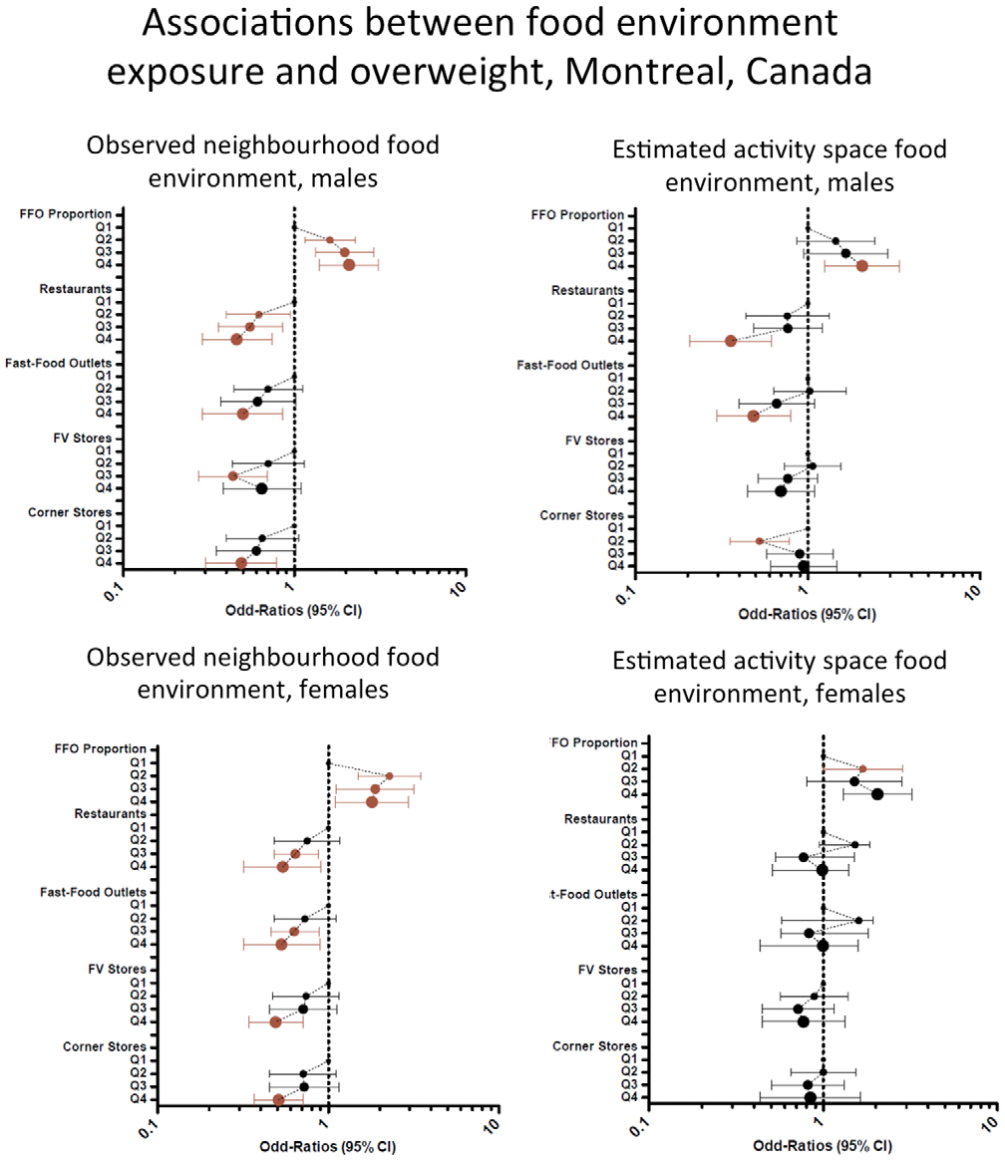
Associations between food environment exposure and overweight, Montreal, Canada.

The strongest associations were observed in Montreal among males with the highest densities of activity space exposure to restaurants (OR = 0.36) and fast food outlets (0.48) (See Figure 2). Similar but however weaker associations were observed at the neighborhood level (OR of 0.46 and 0.50 respectively).

Among women, the strongest reductions in MOR were observed when accounting for the neighborhood proportion of restaurants being fast food outlets, both in Montreal and Quebec City. Living in the neighborhoods with the highest proportions of fast food outlets was associated with a higher risk of being overweight. Yet, crude fast food outlets and full service restaurant densities were negatively associated when considered independently. The direction of the associations were consistant between residential and activity space predictors for a given foodstore type. However, in some cases, only one type of exposure measure was significant.

## Discussion

We undertook a modelling exercise that sought to account for the multiple exposures resulting from daily mobility to help explain how foodstore exposure relate to overweight. The approach combined data on individuals' mobility, foodstore locations and self-reported height and weight. Mobility patterns from travel surveys were used to map participants' activity locations and establish activity space exposure measures to foodstores. Models of such exposure measures were then applied to participants of a health survey (CCHS) living in the same territory. This allowed estimation of activity space exposure to foodstores for individuals for whom only health data and residential location – and no details on their out-of-home activity locations – was initially available. Using CCHS respondents from two survey cycles −2.1 and 3.1- in Montreal and Quebec City, both residential observed and activity space estimated foodstore densities were then tested as predictors of overweight.

A number of important results can be summarized here. First, the high proportion of explained variance when modelling activity space exposures point to the capacity of individual-level and residential characteristics to predict such outcomes. This indicates that although mobility patterns are very much unique to individuals, the foodstore environment that individuals experience can reasonably be predicted based on one's age, occupation, household type, and residential area characteristics.

Second, while accounting for mobility patterns revealed higher average exposures to fast food outlets and full service restaurants than when considering only residential exposures, small differences were observed for corner stores, fruit and vegetable stores and the proportion of restaurants being fast food outlets (See Table 2). This suggests that exposure measures limited to the residential neighborhood may underestimate true exposure to certain foodstore types. Consequently, associations between health measures and environments referenced only to residential locations can be biased, in accordance with previous research showing bias in neighborhood-limited measures of exposure to nitrogen dioxide [31].

Third, activity space exposure estimates were often associated with overweight. Moreover, activity space predictors explained more between neighborhood variance – yielded a higher reduction in the median odd ratio – than residential measures among males, while the contrary was observed among females. Accounting for mobility patterns when estimating exposure could increase our capacity to explain individuals' likelihoods of being overweight while also contributing to explaining geographic differences – i.e. reducing between-neighborhood unexplained variance. The role of activity space measures has been reported recently in a cohort of adults in relation to depression and cervical screening in Paris, France [43], [44]. Although concerning very different outcomes, these findings are together indicative of the potential importance of considering mobilities and non-residential activity locations for epidemiological modeling.

The fact that for women few activity space indicators were significant whereas residential neighborhood measures almost always were is interesting. The precise mechanisms that would explain why the influence of experienced food environments operates in different ‘spaces’ – neighourhood vs. activity space – is yet unclear. Further research is required to better describe and understand mobility patterns and the form and nature of activity spaces. Regarding this specific gender difference, one could wonder if there is a spatial mismatch between male and female workspaces. Are women more often employed in food-related businesses? Could there be an influence linked to lunch diet? Do women have a greater tendency to consume a lunch at the workplace that was prepared at home? Time use surveys report gender differences in time spent on unpaid housework including shopping for food and food preparation, although secular trends have shown a relative convergence between sexes [45]. In 2010, the General Social Survey providing time use data for Canadians showed that among dual-earner couples without children, women spent 14.8 hours per week on domestic work (including shopping for food and meal preparation, but also other domestic tasks such as housecleaning or maintenance and repair), compared to 11.5 hours for men (−22.2%) [46]. Interestingly however, significant differences were also observed between single men and women, the former spending 11.7 hours a week on domestic work compared to 9.7 hours for the latter (−17.1%). Further work is needed to better understand the possible links between gender roles in food-related activities including shopping for food or meal preparation and observed differences in the influence of neighbourhood and activity space exposure to foodstores on overweight. Models accounting for gender, household type as well as occupation could shed light on these questions. Finally, this paper has demonstrated the possibility to combine distinct datasets of travel and health surveys. One could imagine further adding time use survey data to adjust for existing differences in time use regarding food-related domestic activities. Along the same line, considering information on the nature of activities could further help identify activity locations that where visited for food purchase or consumption. Unfortunately, the travel survey used did not provide enough detail on trip purpose to do so.

Although tested with two contrasting cities, the role of large scale city-wide factors remains yet unexplained and residual confounding of the associations observed here is possible. The geographies of overweight differed: in Montreal, a relatively strong residual spatial structure was present for both men and women. No such spatial structure was actually observed for men in Quebec City. Observed residential and activity space food environment exposures were 2 to 4 times stronger in Montreal. Broader city-level dimensions, such as urban sprawl and associated mobility patterns may explain observed differences between cities. Inclusion of different times of measures or further consideration of other contrasting cities and related urban form indicators may help to better understand between-city differences.

### Limitations

A number of limitations need to be acknowledged, and suggest this approach may further be refined for future assessment of activity space exposure and linkage to health outcomes. Limitations relate to the quality and coverage of the survey data used, and to the modelling approach itself.

The mobility data provided information on trips for a single weekday only and was self-reported from the surveyed person for all household members. Activity locations used herehence only represented a limited portion of peoples' true activity space. Yet, because data were available for large numbers of respondents, it can be argued that it provides a representative picture of the types of places people are exposed to. Modelling of traveled distances achieve overall lower explanatory powers than was achieved here in modelling the *types* of exposures people experience, suggesting that although there is strong inter-individual variability in mobility itself, people with similar profiles tend to visit similar *types* of places, or at least places with similar degrees of exposure to foodstores. This can in part be explained by concentration of certain activity places such as workplaces in the Central Business District, which act as ‘spatial hubs’ where people share common exposure attributes.

Other limitations relate to data and methods used to describe the food environment itself [47]. The listing of foodstores was validated on site, and showed good validity [36]. Nevertheless, measuring food environments and assessing their impact on health calls for more than simply looking at accessibility by foodstore type. First, non-spatial concepts of affordability, acceptability, and accommodation may play a role, beyond accessibility and availability [48]. Second, there are various ways to measure accessibility itself. The 29 papers included in a recent review on GIS measures of food accessibility used distance to closest resources or density estimates [47]. All reviewed papers also used one unique observation points per individual to derive measures. Limiting such accessibility measures to people's homes or schools prevents us from exploring the broader dose-response relationship between multiple environmental exposures and health. This paper suggests that it may be important to consider one's “personal network of usual places" [27] to assess accessibility or exposure to environmental risk conditions in multiple locations.

Discrepencies in dates of various datasets also represent a limitation of this paper. Commercial foodstore listings are generally updated continuously, which makes recent datasets relatively easy to obtain. The drawback is that companies only rarely keep backups of older datasets, which makes it difficult to obtain data for retrospective snapshots of the food environment. In our digital era, it is important to keep memories of ‘how things were’, particularly in a context were lifetime exposures may be of relevance.

This leads to another limitation of this paper, which is that temporal aspects of exposure were ignored. All activity locations were weighted equally, without consideration of (i) the amount of time spent at the location, and (ii) time of the day. Exposure could be weighted according to time spent at a given location, and could account for store opening hours, so as to adapt exposure measures for night-shift workers for example. Similarly, if routes between activity locations could be accounted for, exposure could be inversely weighted with speed at which one passes by a ressource. Accessibility measures at a given location could further integrate time budget constraints, if known, in line with space-time geography principles [49] [17].

Finally, the outcome variable overweight was derived from self-reported height and weight, which is known to be biased. Analysis of subsamples of CCHS participants for whom measures of height and weight were available in both self-reported and measured form revealed that self-reported height was overestimated in average (+1 cm for males and +0.5 among females in subsample of cycle 3.1) while self-reported weight was underestimated (−2.5 kg among females and −1.8 kg among males) [50], [51]. Furthermore, differences between self-reported and measured height or weight were more pronounced with increasing BMI. Age has also been associated with underreporting of BMI, yet not in a constant way between cycles. If a subsample of our observation sample had had measured height and weight, one could have established a predictive regression model based on self-reported height and weight and other individual-level characteristics. However, such subsample was not available in either cycles 2.1 or 3.1 which were used in our study. Other limitations relate to the modelling approach. Because the survey providing the mobility data did not contain any health information, and because we could not identify any health survey for which detailed mobility data was available, we inferred mobility and corresponding exposure patterns from participants of travel surveys to participants of health surveys. It was deemed appropriate to transfer the mobility properties – and the related exposure to food environment – of one sample to another because participants of both surveys were covering the same territories, had been interviewed at similar dates, had similar profiles, and because the models of foodstore density exposures explained a relatively high portion of the observed variance. Although this approach seems reasonable, it appears that some predictive models underestimated absolute exposure levels. This was especially true for fast food and full-service restaurants in Montreal, where predicted activity space exposures were significantly lower than observed exposure levels. Yet, it was assumed that relative differences between individuals were maintained, which allowed observing associations between variations in exposure in relation to variations in overweight. The modelling of overweight itself could be improved by including other individual covariates such as lifestyle indicators, or built environment characteristics, while self-reported measures of height and weight also constitute a limitation.

Finally, considering actual activity locations to measure exposure to environments raises a problem of self-selection. Similar to the residential self-selection bias were people may choose their location because of neighborhood characteristics associated to the behaviour of interest – walkable neighbourhoods and walking for example – people choose daily destinations to conduct certain activities that may be directly related to the outcome of interest. For example, people who visit a fast-food restaurant for lunch will be attributed a high level of exposure to fast food restaurant, yet such an exposure measure result from a conscious choice to visit such a destination. In order to reduce such selective daily mobility bias, one may actually remove the destinations that correspond to activities that are directly related to the outcome of interest, and simply retain exposure measures from the other activity location points (Chaix et al. submitted). In our example, exposure to the fast food restaurants would then established from all other ‘non-eating’ activity locations, places at which exposure levels or accessibility could be more or less condusive to the behaviour – eating at a fast food restaurant – and related health outcome of interest – BMI. Yet, in order to be able to handle this self-selection bias, one must know the nature of the activity that was conducted at a given location. Studies are increasingly using mobility data to assess multiple exposures, for example using global positioning system (GPS) trackers. Yet, knowing precise location of people does not translate into exact knowledge of the nature of activities being conducted. Complementary methods can be used to collect such information from the user, through prompted recall surveys [52], [53], [54], Ecological Momentary Assessment techniques [55], or map-based interactive questionnaires [29]. Estimation of the nature of activities can also be based on algorithms processing datastreams from an array of sensors such as accelerometers, cameras or microphones [56], [57]. Increasingly, multisensor architectures are being developed to capitalise on sensors embedded in smartphones [58], [59], [60].

Beyond the discussed limitations, this study demonstrates that it is feasible to use mobility data to assess activity space experienced exposures to food environments. Accounting for multiple exposures can improve our understanding of the dose-response relationship between environments and health outcomes. Use of activity space exposure measures did in certain cases enhance the capacity to explain area-level variation in overweight. This approach is promising because it offers a general modelling framework for improved environmental exposure assessment. Urban areas generally dispose of travel surveys which render this method replicable. As discussed, complementary methods for collecting regular destinations within health surveys are however also required, and would circumvent the limitation of transferring mobility behaviour from one sample to another. Novel approaches, for example using GPS devices [61] or interactive mapping questionnaires [29], provide interesting avenues to help obtain precise information on activities and visited locations. If self-selection bias can be properly addressed, consideration of multiple daily exposuresshould improve our understanding of environmental influences on health, and provide evidence for designing adapted public health interventions.
